# In vitro performance evaluation of AnaConDa^TM^-100 and AnaConDa^TM^-50 compared to a circle breathing system for control and consumption of volatile anaesthetics

**DOI:** 10.1007/s10877-020-00634-4

**Published:** 2020-12-21

**Authors:** Martin Bellgardt, Dominik Drees, Vladimir Vinnikov, Adrian I. Georgevici, Livia Procopiuc, Thomas P. Weber, Andreas Meiser, Jennifer Herzog-Niescery

**Affiliations:** 1 Department of Anaesthesiology and Intensive Care Medicine, Ruhr-University Bochum, St. Josef Hospital, Gudrunstraße 56, 44791 Bochum, Germany; 2 Paediatric Intensive Care Unit, Evelina London Children’s Healthcare, Guy’s and St. Thomas, NHS, Westminster Bridge Road, SE1 7EH London, United Kingdom; 3 Department of Anaesthesiology, Intensive Care Medicine and Pain Medicine, Saarland University Medical Centre, Kirrberger Straße 100, 66424 Homburg/Saar, Germany

**Keywords:** AnaConDa™, Circle breathing system, Costs, Inhalation Anaesthetics, Isoflurane, Sevoflurane

## Abstract

To identify the better volatile anaesthetic delivery system in an intensive care setting, we compared the circle breathing system and two models of reflection systems (AnaConDa™ with a dead space of 100 ml (ACD-100) or 50 ml (ACD-50)). These systems were analysed for the parameters like wash-in, consumption, and wash-out of isoflurane and sevoflurane utilising a test lung model. The test lung was connected to a respirator (circle breathing system: Aisys CS™; ACD-100/50: Puriton Bennett 840). Set parameters were volume-controlled mode, tidal volume-500 ml, respiratory rate-10/min, inspiration time-2 sec, PEEP-5 mbar, and oxygen-21%. Wash-in, consumption, and wash-out were investigated at fresh gas flows of 0.5, 1.0, 2.5, and 5.0 l/min. Anaesthetic target concentrations were 0.5, 1.0, 1.5, 2.0, and 2.5%.  Wash-in was slower in ACD-100/-50 compared to the circle breathing system, except for fresh gas flows of 0.5 and 1.0 l/min. The consumption of isoflurane and sevoflurane in ACD-100 and ACD-50 corresponded to the fresh gas flow of 0.5-1.0 l/min in the circle breathing system. Consumption with ACD-50 was higher in comparison to ACD-100, especially at gas concentrations > 1.5%. Wash-out was quicker in ACD-100/-50 than in the circle breathing system at a fresh gas flow of 0.5 l/min, however, it was longer at all the other flow rates. Wash-out was comparable in ACD-100 and ACD-50. Wash-in and wash-out were generally quicker with the circle breathing system than in ACD-100/-50. However, consumption at 0.5 minimum alveolar concentration was comparable at flows of 0.5 and 1.0 l/min.

## Introduction

There are two methods of administering volatile anaesthetics (VA) in the intensive care unit (ICU): a circle breathing system and reflection. The circle breathing system consists of a Y-piece attached to the in- and expiratory valves, a breathing bag, a CO_2_ absorber, a pressure-relief valve, and a fresh gas supply. The fresh gas flow (FGF) of the circle breathing system influences wash-in and wash-out times and VA consumption [1]. An FGF of < 1 L/min is economic, because it reduces the loss of VA [2]. The main components of the reflection system, AnaConDa™ (ACD; Sedana Medical, Danderyd, Sweden), include the evaporator and a carbon particle filter that reflects the VA. When the original ACD was operated with 0.9% sevoflurane (SEVO), VA consumption corresponded to an FGF of 1.5 L/min in a circle breathing system [3, 4]. The original device had an internal volume of 100 mL (ACD-100), but recently a small-volume ACD was developed with an internal volume of 50 mL (ACD-50). The expected consequence of the smaller size is better carbon dioxide dissipation but reduced VA reflection. While the filter’s performance has improved, VA consumption and its dependency on FGF were never investigated.

We therefore studied ISO and SEVO lung wash-in, wash-out and consumption when administered via a circle breathing system with one of 4 different fresh gas flows, with the ACD-50, or with the ACD-100, all with a 5 L/min ventilation. We hypothesized lung wash-in and wash-out to be faster and agent consumption to be lower with the circle breathing system.

## Methods

This experimental study was performed in a German University Hospital in March 2015 (circle breathing system and ACD-100) and October 2016 (ACD-50), respectively. Due to the nature of the study using a test lung, ethical approval was waived by the Institutional Review Board.

### Experimental setting

**Fig. 1:**
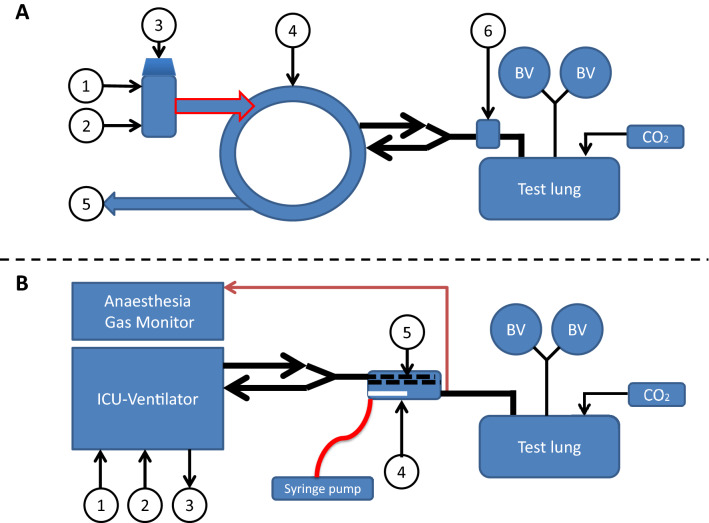
Experimental setup circle breathing system (Aisys CS™ ventilator) and reflection (ACD-50 and ACD-100). **a** Circle breathing system. The setup consists of the circle breathing system (left side) and the test lung (right side). Air (1), oxygen (2), and a vaporized anaesthetic (3) are injected through the anaesthetic machine into the circle breathing system (4). An anaesthesia gas scavenging system is connected (5). The circle breathing system is connected via a heat moisture exchanger (6) to the test lung box with two bag-valve units (BV) with a volume of 2 l each. **b** ACD. Air (1) and oxygen (2) are applied through the Bennett 840 ventilator; an anaesthesia gas scavenging system is connected (3). The Y-piece of the ventilator is attached to the ACD, which consists of an evaporator (4) and a heat moisture exchanger. Gas samples are collected on the patient/test lung side and analysed by a gas monitor. BV = bag-valve unit with a volume of 2 l each

The test lung consisted of a plastic box with a volume of 3.9 L (HPL 829, Lock & Lock, iSi Deutschland GmbH Solingen, Germany), connected to two bag-valve units (volume 2 L each, accessory for Zeus™, Dräger Medical, Lubeck, Germany). The top cover of the box had three openings. The first opening was connected via a Y-piece (6515-12-339-4401, Dräger Medical, Lubeck, Germany) with the two bag-valve units. The second opening was used for carbon dioxide insufflation (AirLiquide Deutschland GmbH, Dusseldorf, Germany). Carbon dioxide concentration was measured on the test lung side and kept between 20 and 40 mmHg. The last opening was connected via a tube elongation (Gänsegurgel 22F-22M/15, P.J. Dahlhausen & Co., Cologne, Germany) with the respective ventilator (Fig. 1). All the experiments were performed under ambient temperature and pressure conditions.

### Circle breathing system

We used the Aisys CS™ anaesthesia ventilator (GE Healthcare, Chalfont St. Giles, GB) with a circle breathing system and automated closed-loop VA delivery. The vaporization of ISO and SEVO was electronically controlled; a VA-specific cassette (Aladin™) was used.

The Aisys CS™ ventilator was connected via a heat moisture exchanger (HME, DAR™, Covidien, Mansfield, MA, USA) with the test lung. An anaesthesia gas scavenging system (AGS, Dräger Medical, Lubeck, Germany) was additionally attached (Fig. 1a). The test lung was ventilated in volume-controlled mode with the decelerating flow (PCV-VG). The respiratory parameters were set as follows: tidal volume 500 ml, respiratory rate 10/min, inspiration time 2 sec, PEEP 5 mbar, and oxygen 21%.

ISO and SEVO were delivered by automated closed-loop control. Wash-in and wash-out times and VA consumption were investigated at four different FGFs (0.5, 1.0, 2.5, and 5.0 L/min) that were manually adjusted (no ‘auto’ mode). These FGFs were each combined with five VA target concentrations (0.5, 1.0, 1.5, 2.0, and 2.5%) for both ISO and SEVO.

As soon as a target concentration was reached, it was kept constant for 30 minutes before VA consumption was documented as displayed by the Aisys CS™ ventilator (the consumption was saved automatically every six seconds; from this, an average value was calculated after 30 minutes). For wash-out, the VA target concentration was set to zero. Times to reach 2.0, 1.5, 1.0, 0.5, and 0% were recorded.

### ACD-50 and ACD-100

The ACD is a modified HME that captures the inhaled agent during exhalation and releases a high portion of it during the next inspiration in a process called anaesthetic reflection. The ACD was set up and primed with liquid ISO or SEVO (25 mL/hr for 2.5 minutes) by a syringe pump (Perfusor® Space, B. Braun Melsungen AG, Melsungen, Germany) according to the instructions of the manufacturer. Once the gas monitor detected the used VA, the syringe pump rate was reduced to 10 mL/hr to avoid a VA overdose. Once the first VA target concentration was reached, the pump rate was manually adjusted to keep the target concentration constant for 30 minutes. The VA consumption was read from the syringe pump. For wash-out, the syringe pump was stopped with the ACD left in place. 200 mL/min gas were collected on the test lung side of the ACD, analysed by a gas monitor (Vamos, Dräger Medical, Lubeck, Germany), and scavenged.

The test lung was ventilated with a Puriton Bennett 840 ventilator (PB-840, Medtronics, Minneapolis, USA) was used, which was connected to an anaesthesia gas scavenging system (AGS, Dräger Medical, Lubeck, Germany) (Fig. 1b). The test lung was ventilated in volume-controlled mode with decelerating flow pattern (SIMV-Volume Control plus). Ventilation parameters, FGFs (0.5, 1.0, 2.5, and 5.0 L/min), and VA target concentrations (0.5, 1.0, 1.5, 2.0, and 2.5%) were the same as described for the circle breathing system.

All the tests were repeated three times.

## Statistics

The sample size calculation was based on an experimental study by Bomberg et al., who investigated ISO concentrations (0.5–20 ml/h) in a test lung using ACD-50 and ACD-100 (n = 3) [7]. Due to the low amount of error-related variance in this setting, effect sizes were remarkably high (d: 44.42 ± 49.07; minimum: 1, maximum: 105.93). Given the above results, a conservative, but still high effect size of d = 4, a power of 0.8, and an alpha of 0.05 would result in 3 repetitions of each condition to investigate the targeted effects in a test lung (independent t-test ACD-50 and ACD-100 vs. circle breathing system, FGFs 0.5–2.5 l/min).

Mixed model Analysis of Variance (ANOVA) with the ‘between’ factor *device* (ACD-50/ACD-100 vs. circle breathing system, FGF 0.5, 1.0, 2.5, 5.0 l/min), and the ‘within’ factors *time to targeted concentration* during wash-in and *expiratory VA concentration* during consumption (both with expiratory VA concentrations of 0.5, 1.0, 1.5, 2.0, and 2.5%) were conducted separately for ISO and SEVO. In case of a significant interaction, a univariate ANOVA for each level of the ‘within’ factor was performed to investigate differences between ACD and circle breathing system. Both ACD-50 and ACD-100 were tested separately and used as reference conditions in the follow-up analyses via Dunnett T-Test.

Differences between the ACD and the circle breathing system regarding wash-out from 2.5 to 0% were tested via univariate ANOVA, modelling the ‘between’ factor *device* (ACD vs. circle breathing system: 0.5, 1.0, 2.5, 5.0 l/min FGF) separately for ACD-50 and ACD-100. In case of a significant main effect, follow-up tests were performed via Dunnett-T-Tests with the ACD as a reference category.

To test for differences between ACD-50 and ACD-100 during wash-in and consumption, mixed-model-ANOVAs were performed as stated above. Post-hoc-analyses during wash-in and consumption, as well as the test for differences regarding wash-out were conducted via independent t-tests, as there was no reference category.

Adjustments for multiple comparisons were performed via Bonferroni-correction.

Effect sizes are reported for significant effects on ANOVA-level via Eta^2^_p_ (with 0.01 displaying a small effect, 0.06 displaying a medium effect, and 0.14 displaying a strong effect), and on t-test-level (with r = 0.1 / 0.3 / 0.5, respectively).

In this study, statistical analyses were understood as a rough estimation of the real effect, as the sample size was low. However, the error-related variance is low in this model-based experimental setting.

## Results

### VA wash-in

#### Circle breathing system 

**Fig. 2:**
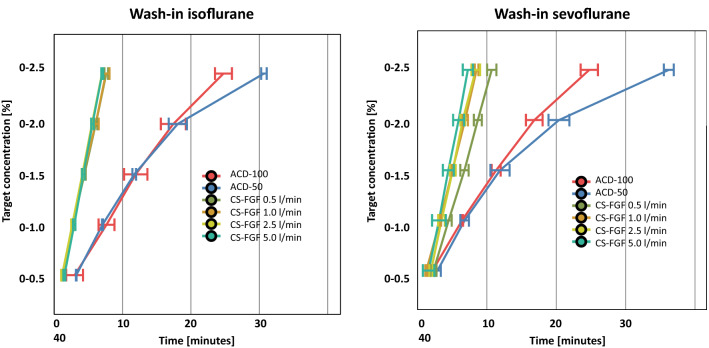
*Wash-in of ISO und SEVO*. In the circle breathing system (CS) the time to reach the set expiratory anaesthetic gas concentration depended on the height of the target concentration but was independent of the fresh gas flow (FGF). Both ACD devices needed significantly longer to reach any target concentration than the CS, except for ISO at 0.5%. Thereby, the performance of the ACD-50 and ACD-100 were comparable for target concentrations up to 2.0%, but the ACD-50 took significantly longer to reach 2.5%. Error bars represent ± 1 standard deviation

The wash-in of ISO and SEVO was not significantly different throughout all measurements within the circle breathing system and thus did not depend on the FGF (p > 0.05) (Fig. 2).

#### ACD-50 vs. circle breathing system

ACD-50 ISO wash-in always took longer than the circle breathing system, independent of the used VA, the targeted VA concentration, and the set rates of FGF (all p < 0.001) (Fig. 2; Table 1). Differences increased with increasing VA target concentrations (Fig. 2).

**Table 1:** Wash-in for isoflurane and sevoflurane

	0–0.5%	0–1.0%	0–1.5%	0–2.0%	0–2.5%	Ref: ACD-50	Ref: ACD-100
Wash-in Isoflurane [min:sec]							
CS FGF 0.5 l/min	01:14 ± 00:04	02:45 ± 00:05	04:25 ± 00:11	06:06 ± 00:13	07:38 ± 00:16	p < 0.001	p < 0.001
CS FGF 1.0 l/min	01:10 ± 00:06	02:42 ± 00:06	04:14 ± 00:11	06:05 ± 05:32	07:34 ± 00:32	p < 0.001	p < 0.001
CS FGF 2.5 l/min	01:11 ± 00:09	02:38 ± 00:07	04:07 ± 00:04	05:32 ± 00:07	07:00 ± 00:05	p < 0.001	p < 0.001
CS FGF 5.0 l/min	01:30 ± 00:09	02:56 ± 00:10	04:16 ± 00:10	05:37 ± 00:10	07:02 ± 00:08	p < 0.001	p < 0.001
Wash-in Sevoflurane [min:sec]							
CS FGF 0.5 l/min	02:18 ± 00:25	04:29 ± 00:26	06:46 ± 00:36	08:41 ± 00:34	10:41 ± 00:40	–	–
CS FGF 1.0 l/min	01:15 ± 00:07	03:15 ± 00:08	05:02 ± 00:09	06:51 ± 00:24	08:45 ± 00:27	p < 0.001*	p < 0.001**#**
CS FGF 2.5 l/min	01:57 ± 00:15	03:27 ± 00:29	05:04 ± 00:25	06:31 ± 00:20	08:06 ± 00:18	p < 0.001	p < 0.001
CS FGF 5.0 l/min	01:35 ± 00:49	03:05 ± 00:59	07:16 ± 00:47	05:55 ± 00:47	07:15 ± 00:44	p < 0.001	p < 0.001

Data are presented as mean ± 1 standard deviation for each condition. P-values display Dunnetts-T Test, in which ACD-50 and ACD-100 were chosen as reference categories. The main effect *device* of the univariate ANOVA is reported as first-level post-hoc analysis for the mixed-model ANOVA; Dunnett’s T is reported as a second-level post-hoc test within each univariate ANOVA

*p for 0–1% (ACD-50) wash-in is 0.004

#p for 0–1% (ACD-100) wash-in is 0.007

* ACD*  AnaConDa™,* CS*  circle breathing system,* FGF* fresh gas flow

#### ACD-100 vs. circle breathing system 

The ACD-100 required significantly longer time to reach the ISO target concentration than the circle breathing system (p < 0.001), but 0.5% ISO was an exception, as it was comparable in both devices. A similar result was found for SEVO. The ACD-100 took significantly longer to reach the target concentration than the circle breathing system, except for 0.5% SEVO. Here, no statistical difference was seen (Fig. 2; Table 1).

#### ACD-50 vs. ACD-100

Comparing ISO wash-in times for ACD-50 and ACD-100 across the different target concentrations, data revealed no differences for 0.5, 1.0, 1.5, and 2.0%, however, the ACD-50 required roughly six minutes more to reach 2.5% compared to ACD-100. A comparable observation was made for SEVO: no difference up to concentrations of 2% were seen, however, the ACD-50 took about 12.5 minutes longer to reach 2.5% than the ACD-100 (Fig. 2; Table 2).

**Table 2:** Wash-in for isoflurane and sevoflurane

Time to target concentration	ACD-100 [min:sec]	ACD-50 [min:sec]	p-value
Wash-in Isoflurane			
0–0.5%	03:00 ± 01:13	03:14 ± 00:02	0.773
0–1.0%	07:40 ± 01:09	07:04 ± 00:04	0.471
0–1.5%	11:56 ± 01:42	11:43 ± 00:16	0.843
0–2.0%	17:26 ± 01:49	18:05 ± 01:19	0.644
0–2.5%	24:45 ± 01:14	30:40 ± 00:24	0.001
Wash-in Sevoflurane			
0–0.5%	02:09 ± 00:18	02:52 ± 00:28	0.093
0–1.0%	06:25 ± 00:08	06:47 ± 00:37	0.041
0–1.5%	11:20 ± 00:39	11:53 ± 01:22	0.572
0–2.0%	16:49 ± 01:11	20:23 ± 01:30	0.033
0–2.5%	23:55 ± 02:30	36:13 ± 00:42	0.001

Data are presented as mean ± 1 standard deviation for each condition. P-values display Dunnetts-T Test; Dunnett’s T is reported as a second-level post-hoc test within each univariate ANOVA

*ACD*  AnaConDa™,* CS*  circle breathing system,* FGF* fresh gas flow

### VA consumption

#### ACD-50 vs. circle breathing system

**Fig. 3:**
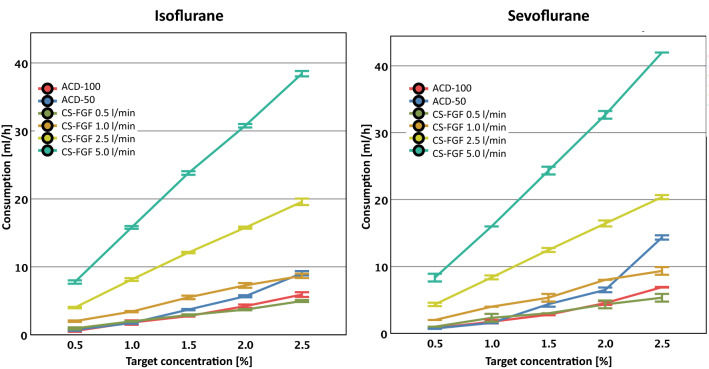
Consumption of ISO and SEVO after wash-in. Up to 2% the anaesthetic gas consumption of both ACD devices corresponded to a fresh gas flow (FGF) < l/min of the circle breathing system (CS), whereas it was about 1 l/min at 2.5%. The ACD-50 showed a significantly higher consumption than the ACD-100 for 2.0% and 2.5%. Error bars represent ± 1 standard deviation

The ISO consumption of the ACD-50 at VA target concentrations of 0.5 and 1.0% corresponded to an FGF of 0.5 L/min in the circle breathing system. At 1.5 and 2.0%, ISO consumption via ACD-50 was comparable to an FGF of 0.5–1.0 L/min, and 2.5% corresponded to an FGF of 1 L/min (Fig. 3; Table 3).

**Table 3:** All authors read and approved the final manuscript

	0.5%	1.0%	1.5%	2.0%	2.5%	Ref: ACD-50	Ref: ACD-100
Consumption Isoflurane [ml/h]							
CS FGF 0.5 l/min	0.93 ± 0.15	1.97 ± 0.06	2.90 ± 0.10	3.70 ± 0.10	4.97 ± 0.15	0.5%: p = 0.405 1.0%: p = 0.229 1.5%: p = 0.001 2.0%, 2.5%: p < 0.001	0.5%: p = 0.037 1.0%: p = 0.684 1.5%: p = 0.727 2.0%: p = 0.075 2.5%: p = 0.026
CS FGF 1.0 l/min	2.00 ± 0.10	3.40 ± 0.10	5.50 ± 0.26	7.27 ± 0.35	8.67 ± 0.32	0.5- 2.0%: p < 0.001 2.5%: p = 0.473	0.5- 2.0%: p < 0.001 2.5%: p = 0.026
CS FGF 2.5 l/min	4.00 ± 0.10	8.13 ± 0.21	12.10 ± 0.10	15.77 ± 0.15	19.57 ± 0.47	p < 0.001	p < 0.001
CS FGF 5.0 l/min	7.77 ± 0.25	15.80 ± 0.20	23.80 ± 0.26	30.77 ± 0.25	38.43 ± 0.40	p < 0.001	p < 0.001
Consumption Sevoflurane [ml/h]							
CS FGF 0.5 l/min	1.00 ± 0.00	2.33 ± 0.58	3.00 ± 0.00	4.33 ± 0.58	5.33 ± 0.58	0.5%: p = 0.620 1.0%: p = 0.028 1.5%: p = 0.009 2.0%, 2.5%: p < 0.001	0.5%: p = 0.729 1.0%: p = 0.125 1.5%: p = 0.912 2.0%: p = 0.943 2.5%: p = 0.002
CS FGF 1.0 l/min	2.00 ± 0.00	4.00 ± 0.00	5.33 ± 0.58	8.00 ± 0.00	9.33 ± 0.58	0.5%: p = 0.001 1.0%: p < 0.001 1.5%: p = 0.059 2.0%: p = 0.007 2.5%: p < 0.001	0.5%: p = 0.001 1.0-2.5%: p < 0.001
CS FGF 2.5 l/min	4.33 ± 0.25	8.37 ± 0.29	12.47 ± 0.31	16.43 ± 0.45	20.37 ± 0.31	p < 0.001	p < 0.001
CS FGF 5.0 l/min	8.33 ± 0.58	16.00 ± 0.00	24.33 ± 0.58	32.67 ± 0.58	42.00 ± 0.00	p < 0.001	p < 0.001

Data are presented as mean ± 1 standard deviation for each condition. P-values display Dunnetts-T Tests, in which ACD-50 and ACD-100 were chosen as reference categories.

*ACD*  AnaConDa™,* CS*  circle breathing system,* FGF*  fresh gas flow

The same observations were made for SEVO. The consumption of the ACD-50 corresponded to an FGF of 0.5 L/min at 0.5% and 1.0%, and 1.0 L/min at 1.5%. A comparison of SEVO consumption between the circle breathing system at all tested FGFs and the ACD-50 revealed a statistically significant difference at 2.0% (p ≤ 0.007) and 2.5% (p < 0.001), indicating that no corresponding FGF for these target concentrations could be mentioned (Fig. 3; Table 3).

#### ACD-100 vs. circle breathing system 

The consumption of the ACD-100 was comparable to an FGF of 0.5 L/min in the circle breathing system for ISO and SEVO. An exception was 2.5% SEVO: here, the consumption was slightly higher than the FGF of 0.5 L/min of the circle breathing system (Table 3).

#### ACD-50 vs. ACD-100

ACD-50 showed a significantly higher ISO-consumption than ACD-100 for 1.5%, 2.0%, and 2.5%. There were no differences at 0.5% and 1.0%.

For SEVO no differences were seen until 1.5%, but the ACD-50 had shown a significantly higher consumption than the ACD-100 for 2.0% and 2.5% (Table 4).

**Table 4:** Consumption of isoflurane and sevoflurane

Consumption at target concentration	ACD-100 [ml/h]	ACD-50 [ml/h]	p-value
Consumption Isoflurane			
0.5%	0.55 ± 0.11	0.73 ± 0.15	0.161
1.0%	1.79 ± 0.33	1.73 ± 0.12	0.776
1.5%	2.75 ± 0.08	3.70 ± 0.10	< 0.001
2.0%	4.21 ± 0.25	5.67 ± 0.15	0.001
2.5%	5.92 ± 0.34	9.07 ± 0.32	< 0.001
Consumption Sevoflurane			
0.5%	0.77 ± 0.03	0.73 ± 0.06	0.366
1.0%	1.75 ± 0.25	1.57 ± 0.06	0.291
1.5%	2.79 ± 0.08	4.37 ± 0.38	0.015
2.0%	4.53 ± 0.28	6.50 ± 0.35	0.002
2.5%	6.91 ± 0.04	14.33 ± 0.32	0.001

Data are presented as mean ± 1 standard deviation for each condition. P-values display Dunnetts-T Tests

*ACD* AnaConDa™, * CS*  circle breathing system,* FGF* fresh gas flow

### VA wash-out

#### ACD-50 vs. circle breathing system

ISO wash-out from 2.5 to 0% was shorter for the ACD-50 compared to the circle breathing system at an FGF of 0.5 l/min, however, it was longer at any other FGF.

SEVO wash-out of the ACD-50 was comparable to the circle breathing system at an FGF of 0.5 l/min, and longer at any of the other FGF (Table 5).

**Table 5:** Wash-out for ISO and SEVO

	Wash-out					
	Isoflurane [h:min:sec]			Sevoflurane [h:min:sec]		
	2.5–0%	Ref: ACD 50	Ref: ACD 100	2.5–0%	Ref: ACD 50	Ref: ACD100
ACD-100	1:25:53 ± 0:03:08	p = 0.528	–	1:25:00 ± 0:05:00	p = 0.117	–
ACD-50	1:22:43 ± 0:07:18	–	p = 0.528	1:18:27 ± 0:02:41	–	p = 0.117
CS FGF 0.5 l/min	1:36:36 ± 0:02:53	p = 0.003	p < 0.001	1:23:32 ± 0:03:30	p = 0.272	p = 0.070
CS FGF 1.0 l/min	0:48:43 ± 0:01:15	p < 0.001	p < 0.001	0:50:40 ± 0:06:44	p < 0.001	p < 0.001
CS FGF 2.5 l/min	0:21:17 ± 0:00:34	p < 0.001	p < 0.001	0:20:54 ± 0:00:30	p < 0.001	p < 0.001
CS FGF 5.0 l/min	0:10:44 ± 0:00:09	p < 0.001	p < 0.001	0:12:02 ± 0:00:50	p < 0.001	p < 0.001

Data are presented as mean ± 1 standard deviation for each condition. P-values are derived from Dunnet-T Test, which was used as a post-hoc test in case of a significant main effect *device* within the univariate ANOVA

*ACD*  AnaConDa™,* CS*  circle breathing system,* FGF*  fresh gas flow

#### ACD-100 vs. circle breathing system

ISO wash-out was significantly shorter for the ACD-100 compared to the circle breathing system at an FGF of 0.5 l/min, however, it was longer at any other FGF.

SEVO wash-out was comparable in the ACD-100 and the circle breathing system at an FGF of 0.5 l/min, but longer at any of the other FGF (Table 5).

#### ACD-50 vs. ACD-100

Wash-out of ISO and SEVO was not statistically different between ACD-50 and ACD-100 (Table 5).

## Discussion

In this study, we focused on wash-in, consumption, and wash-out of ISO and SEVO in ACD-100 and ACD-50 devices compared with the circle breathing system. Furthermore, differences between ACD-100 and ACD-50 were evaluated.

Data showed that the time to reach the target concentration did not depend on the FGF in the circle breathing system. At first instance, this observation seems unusual because flow-dependent wash-in times have been described for different anaesthetic machines by prior researchers and this further corresponds to the perception of anaesthetists in clinical practice [5, 6]. However, it is known that the Aisys CS™ anaesthesia ventilator with the VA-specific Aladin™ cassette controls the VA output independent of the manually adjusted FGF. This is done by a proportional valve, which regulates the amount of FGF passing through the cassette. This technique resulted in a significantly quicker wash-in compared to both reflection systems in this lung model. Interestingly, Lucangelo et al. have demonstrated that further quicker wash-in by anaesthetic machines with automated closed-loop delivery could be achieved in patients. They reported an average time of 145 seconds to reach a SEVO concentration of 1.0% with an FGF of 1 l/min (compared to about 240 seconds in this in vitro study) [7].

Wash-in was slower for both ACD-systems than for the circle breathing system, and this was more pronounced with VA concentrations over 2%. This could be explained by the spillover effect, described by Meiser et al. in 2009, or due to a limited gas reflecting mass capacity [8]. However, delivery rates of the ACD-devices were set manually, and it required more than five adjustments on an average until the target concentration was reached. Thus, the slower wash-in of both ACD-systems were certainly influenced by the methodology of the study, which was based on the rather cautious recommendations of the manufacturer to prevent VA-overdoses [7].

The requirement of ISO and SEVO by the circle breathing system increases with increasing FGF and VA concentration. However, this is independent of the respiratory minute volume. In contrast, the consumption in both reflection systems increased with the respiratory minute volume [9]. This is interesting for patients with respiratory failure, as higher respiratory minute volumes are usually necessitated. However, the consumption for 0.5 minimum alveolar concentration (MAC) (corresponds to 0.5% ISO and 1.0% SEVO) via ACD with FGFs between 0.5 and 1.0 l/min was comparable with the consumption of a circle breathing system with automated closed-loop delivery. Higher VA concentrations increased the consumption of VA in all of the tested systems, however, most impressively in both ACD-devices. The ACD-50 is of special interest, as the consumption was highest. This can be explained by the reduction of dead space, which goes along with a reduced reflective mass. Consequently, it is the most cost-effective to use a circle breathing system in patients with high respiratory minute volume and who require deep sedation, followed by ACD-100 and ACD-50. Furthermore, it should be kept in mind that ISO is the cheapest VA [10].

Wash-out was mostly shortest with the circle breathing system. This is as expected because wash-out of the ACD is (in contrast to the circle breathing system) passive only. As the VA concentration drops exponentially, even low VA concentrations may result in sedation for hours (the ACD reflector is highly effective at concentrations between 0.1–0.3%) [11]. However, wash-out can be sped up by removing the reflector and replacing it with an HME [12].

Evaluating the parameters utilising an in vitro lung model was the main limitation, as the observations may differ in a clinical setting in patients. In this context it should be noted that the test lung had a total volume of 7.9 liters, but mechanically ventilated patients with pulmonary diseases regularly have functional residual capacities of less than two liters only [13]. Thus, wash-in and wash-out times are probably too long in this experimental setting due to the larger volume of the lung model. Furthermore, the usage of a constant respiratory minute volume of 5 l/min, although this is probably too low for critically ill patients is the other limitation. Though this was done due to technical reasons, as the comparability of the systems is limited.

## Conclusions

In this lung model VA delivery, wash-in and wash-out were, in general, quicker with a circle breathing system and automated closed-loop VA delivery in comparison to ACD-100 and ACD-50. However, VA consumption for optimal intensive care unit target of 0.5 MAC, was comparable at FGFs of 0.5 and 1.0 l/min, though the patient’s respiratory minute volume must be kept in mind. Clinical studies are needed to investigate the use of circle breathing systems in the intensive care unit.
